# Supplementary material for: Functional exploration of colorectal cancer genomes using Drosophila
Source: Nat Commun. 2016 Nov 29;7:13615. doi: 10.1038/ncomms13615 (PMC5141297; doi:10.1038/ncomms13615)
Supplement: Supplementary Information — Supplementary Figures 1-8 and Supplementary Tables 1-2. [file ncomms13615-s1.pdf]

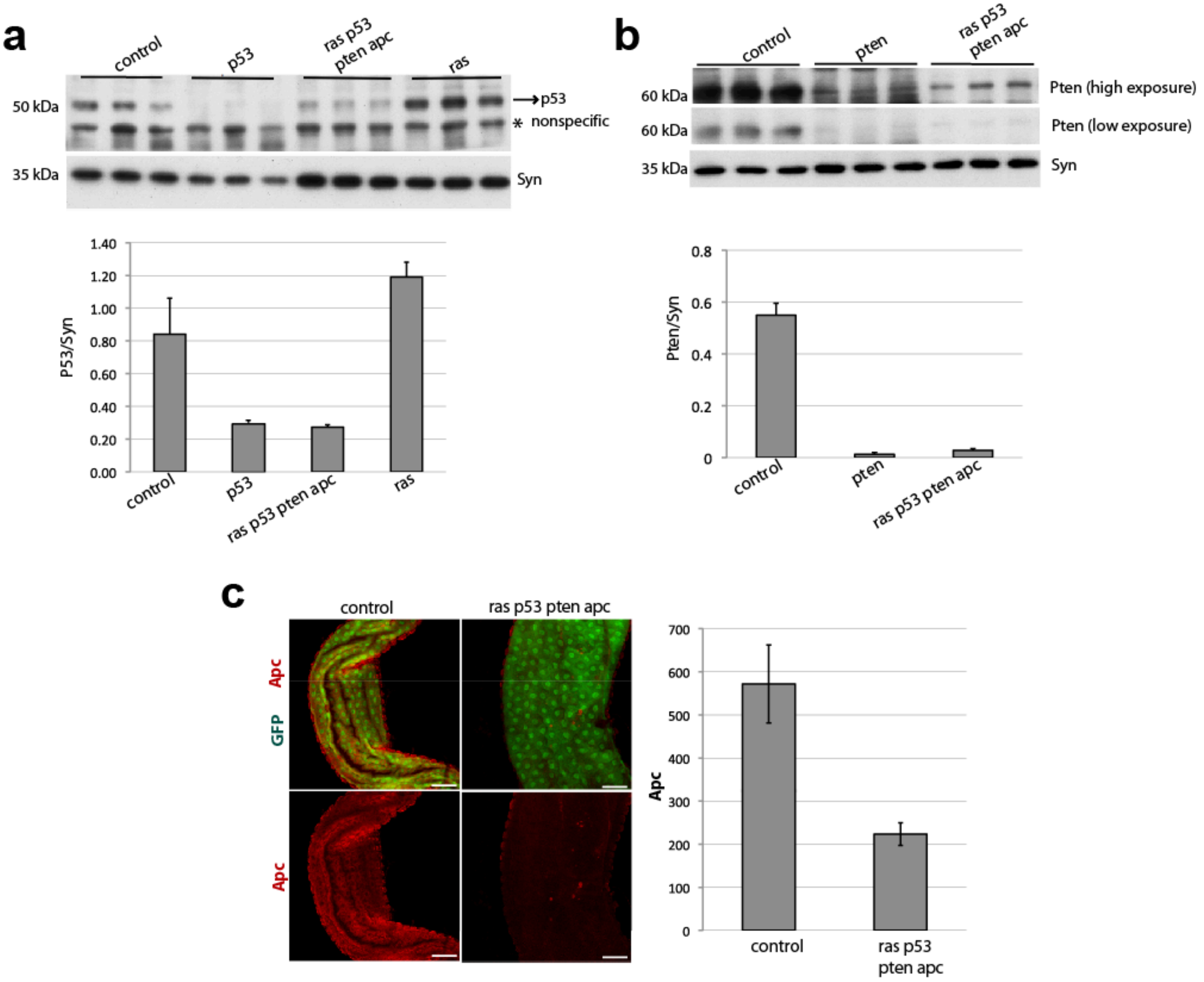

Supplementary Figure 1. Analysis of the efficiency of knock-down in *ras*^{v12} *p53*^{Ri} *pten*^{Ri} *apc*^{Ri}. Analysis of p53 and pten knock-down efficiency in *ras*^{v12} *p53*^{Ri} *pten*^{Ri} *apc*^{Ri} by western blot **(a,b)** and *apc* knock-down efficiency by immunohistochemistry **(c)**. Scale bars: 25 μm. Error bars: standard error of the mean. Uncropped gels used to generate panels **a** and **b** can be found in Supplementary Figure 8g,h.

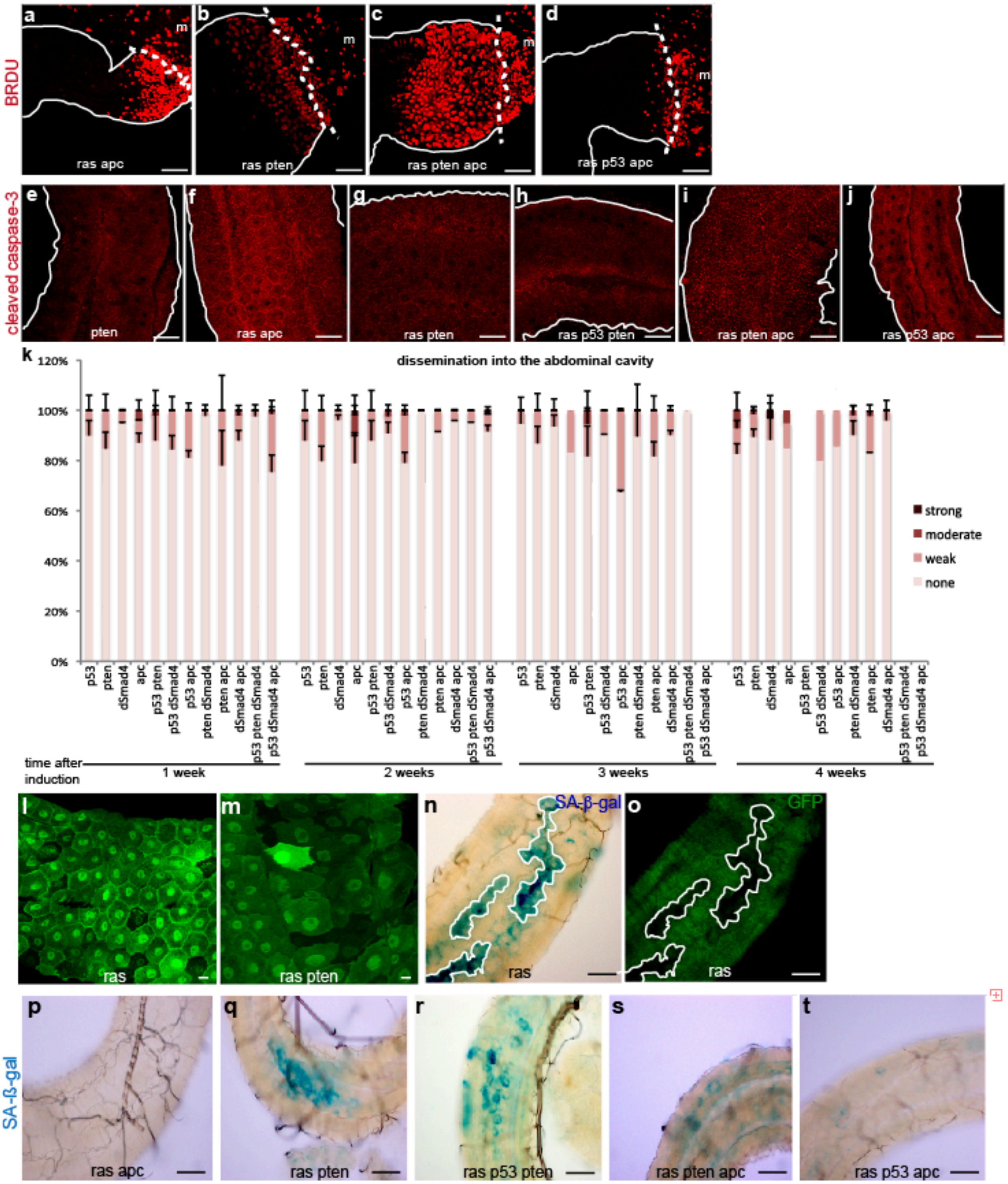

Supplementary Figure 2. Follow-up analyses of cancer phenotypes observed in *ras^{V12} p53^{Ri} pten^{Ri} apc^{Ri}*. a-d, 7 day continuous BRDU labeling (red) of hindguts with the indicated genotypes. Pylorus region of the hindguts are outlined with solid lines; dashed lines indicate hindgut/midgut boundary (m: midgut). **e-j,** Cleaved caspase-3 staining of hindguts (outlined by solid lines) with indicated genotypes. Hindgut cells occasionally displayed high levels of membrane-associated cleaved caspase (e.g. **f**), though its functional significance is unclear. **k,** Quantification of dissemination in animals with indicated genotypes 1, 2, 3 and 4 weeks after induction (n=25-30 flies/replicate; error bars: standard error of the mean). **l, m,** Examples of GFP negative cells from hindguts carrying *ras^{V12}* **(l)** and *ras^{V12} pten^{Ri}* **(m)**. **n,** SA-β-gal positive cells in hindguts carrying *ras^{V12}* were also GFP negative **(o)**. **p-t**, SA-β-gal staining of hindguts with indicated genotypes. Scale bars: 25 μm

a

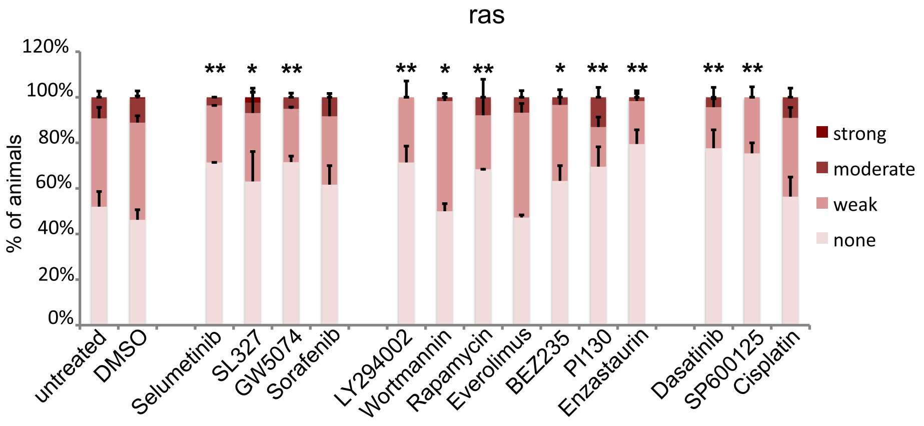

b

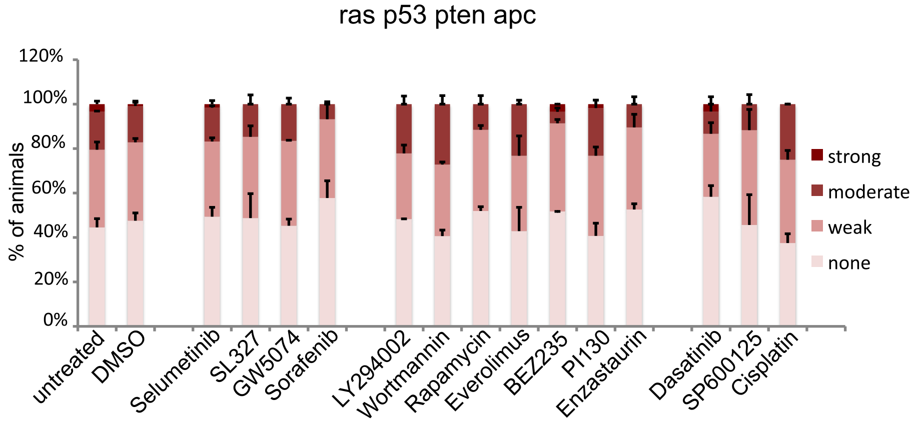

c

	Compound	Target/Mechanism of action	Concentration in the food (mM)	estimated amount ingested/day (ng)*	mg/kg body weight/day*
1	AZD6244	MEK inhibitor	1	92	0.08
2	SL327	MEK inhibitor	1	68	0.06
3	GW5074	Raf inhibitor	0.5	52	0.05
4	Sorafenib	Tyrosine Kinase inhibitor	1	127	0.11
5	LY294002	Dual mTor-PI3K inhibitor	1	61	0.05
6	Wortmannin	PI3K inhibitor	1	86	0.08
7	Rapamycin	mTor inhibitor	1	183	0.16
8	Everolimus	mTor inhibitor	0.5	96	0.08
9	BEZ235	Dual mTor-PI3K inhibitor	0.1	9	0.01
10	PI-103 HCl	Dual mTor-PI3K inhibitor	0.5	41	0.04
11	Enzastaurin	PKC beta inhibitor, also blocks Akt phosphorylation	0.25	26	0.02
12	Dasatinib	Multikinase Inhibitor	1	98	0.09
13	SP600125	Multikinase Inhibitor	1	44	0.04
14	Cisplatin	DNA intercalator	1	60	0.05
15	Bortezomib	proteasome inhibitor	0.01	0.8	0.008
16	Panobinostat	HDAC inhibitor	1	70	0.06

*: calculations based on the following measurements: Average amount of food consumed/fly is ~0.2 μl.
Averege body weight of a fly is 1.1mg

d

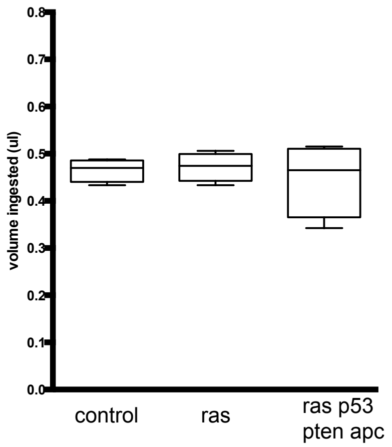

Supplementary Figure 3. Drug response of *ras*^{V12} alone and *ras*^{V12} *p53*^{Ri} *pten*^{Ri} *apc*^{Ri} animals. **a,b,** Quantification of dissemination in *ras*^{V12} **(a)** and *ras*^{V12} *p53*^{Ri} *pten*^{Ri} *apc*^{Ri} **(b)** animals treated with indicated compounds. **c,** List of compounds used in feeding experiments along with their targets, mechanisms of actions and concentrations used in the food. Estimated amount ingested was calculated based on our observation that adult females ingest approximately 0.2 μl of food per day. Estimated amount of ingested compound was also converted into mg/kg body weight/day using 1.1 mg as the average weight of an adult female. **d,** Quantification of amount of food ingested by indicated genotypes. Measurements were done for a period of 8 hours, 4 days after induction of transgenes using the capillary feeding assay (CAFE). **a,b,** n=30 flies/replicate; error bars: standard error of the mean *: $p<0.01$; **: $p<0.05$ (Fisher's exact test). Drug doses indicated reflect concentrations in the food.

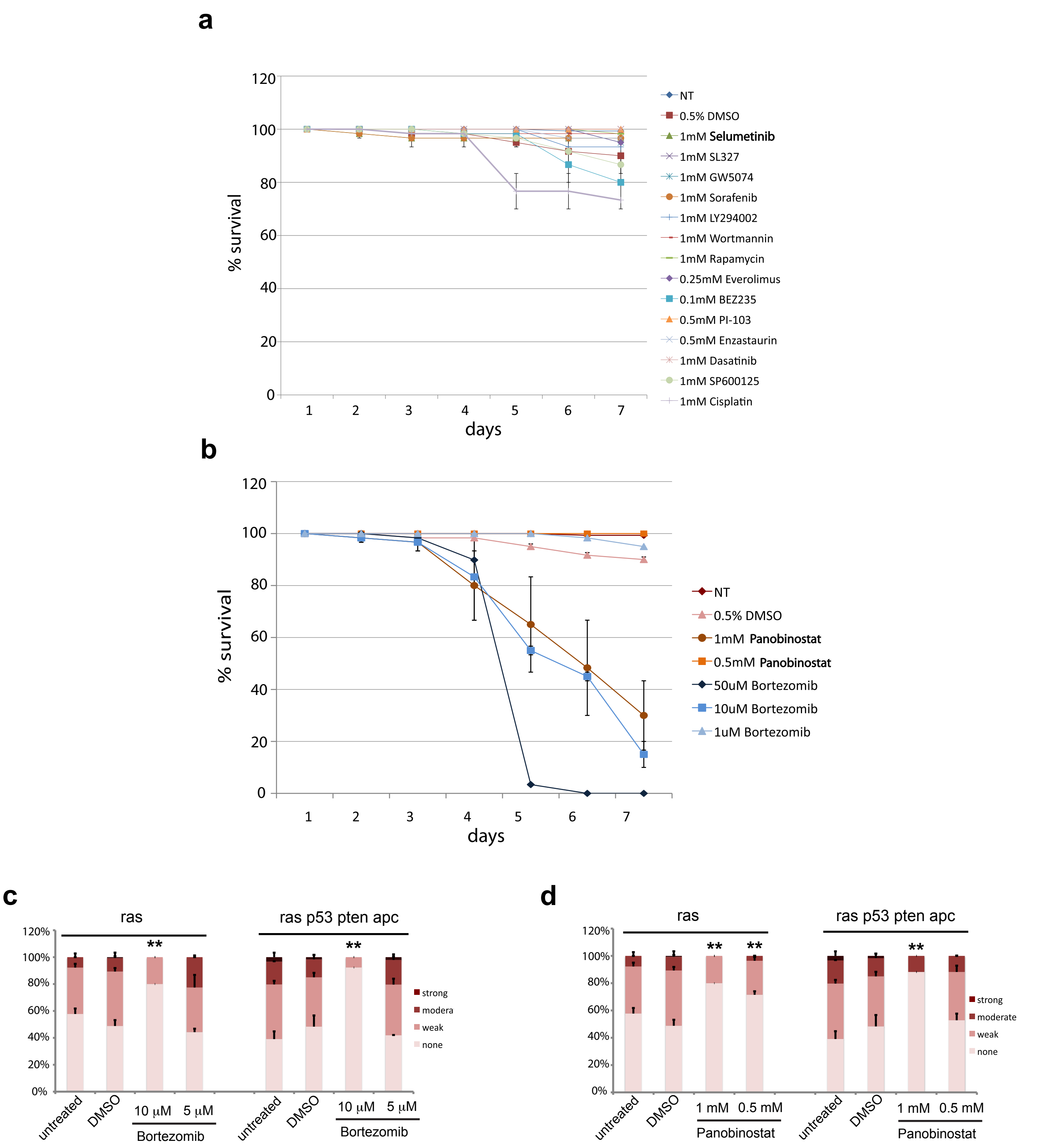

Supplementary Figure 4. Toxicity and therapeutic window for compounds. a, Survival curves of *ras*^{V12} *p53*^{Ri} *pten*^{Ri} *apc*^{Ri} animals fed indicated compounds. At the doses used in our experiments, compounds did not promote significant toxicity except for panobinostat and bortezomib. **b,** Survival curves of *ras*^{V12} *p53*^{Ri} *pten*^{Ri} *apc*^{Ri} animals fed panobinostat or bortezomib at indicated doses. **c,d,** Quantification of dissemination phenotype of animals with indicated genotypes after feeding different doses of bortezomib **(c)** and panobinostat **(d)**. Bortezomib was not effective against either genotype at non-toxic doses (5 µM in the food) while panobinostat was effective only against *ras*^{V12} alone (500 µM in the food). n=30 flies/replicate; error bars: standard error of the mean *: p<0.01; **: p<0.05 (Fisher's exact test). Drug doses indicated reflect concentrations in the food.

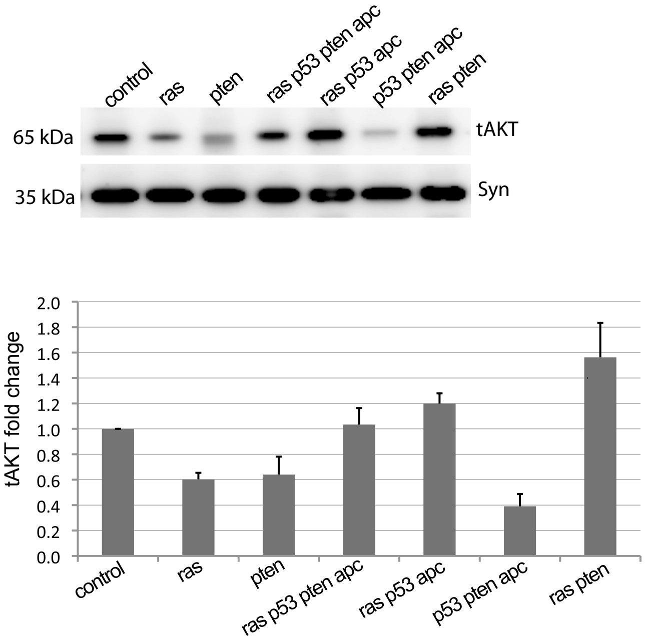

Supplementary Figure 5. Total AKT levels in multigenic combinations. Western blot analysis and quantification of total AKT (tAKT) from hindguts with indicated genotypes. Each data point represents the average response of 2-5 biological replicates with 10 hindguts/replicate. Error bars: standard error of the mean. Uncropped gels used to generate this panel can be found in Supplementary Figure 8j i.

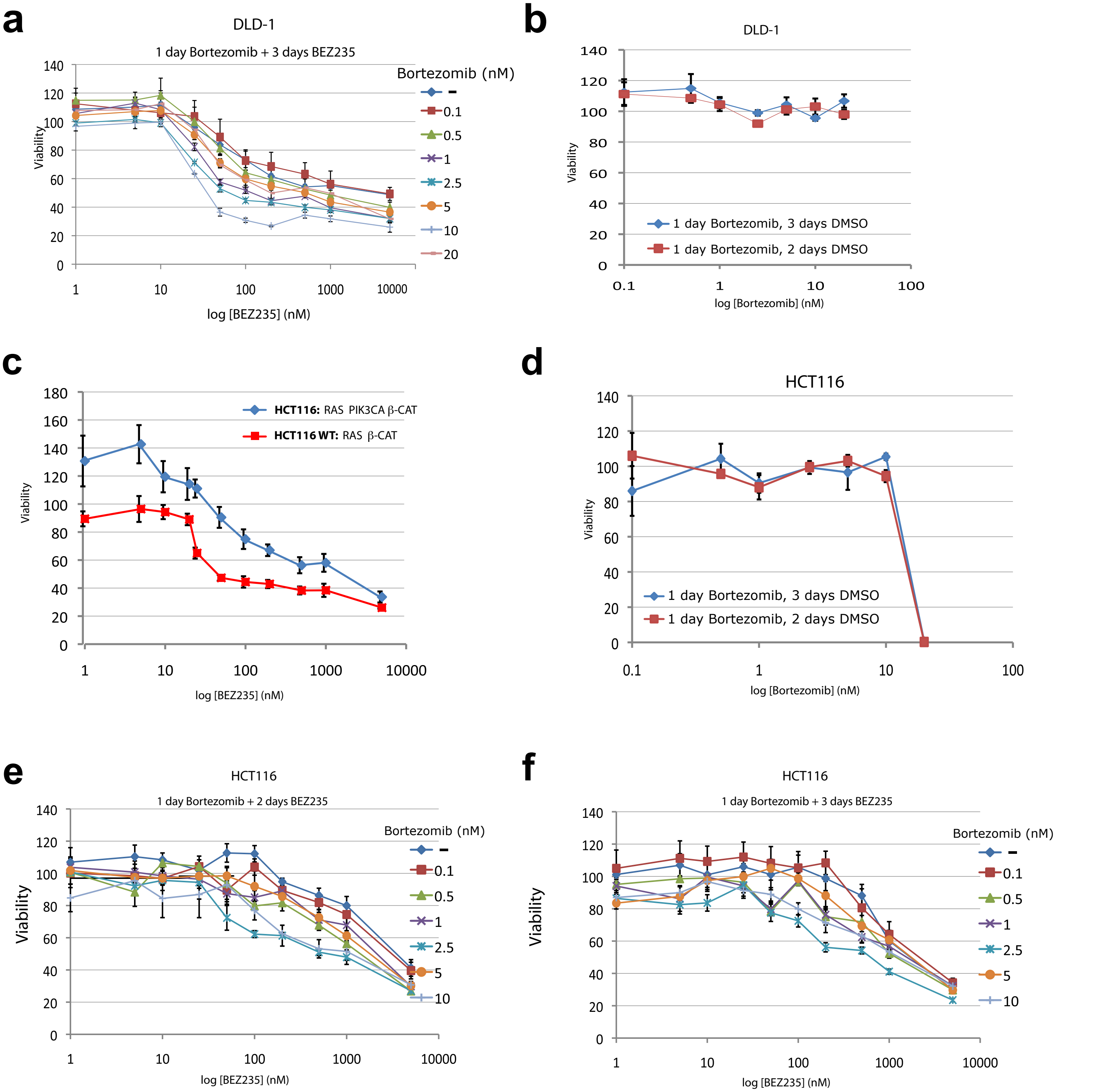

Supplementary Figure 6. Increased sensitivity of DLD-1 and HCT116 cells to BEZ235 after pre-treatment with bortezomib. a, BEZ235 dose response curve after 3 days of BEZ235 treatment of DLD-1 cells that are pretreated with DMSO or indicated doses of bortezomib for 1 day. **b,** Bortezomib pre-treatment alone at the doses used for sequential treatment did not affect the viability of DLD-1 cells. **c,** BEZ235 dose response curve of HCT116 parental (Ras and PI3K active) versus HCT116-WT (Ras active, PI3K wildtype) cell lines. **d,** Bortezomib pre-treatment alone at the doses used for sequential treatment did not affect the viability of HCT116 cells. **e,f,** BEZ235 dose response curve after 2 **(e)** and 3 **(f)** days of treatment of HCT116 cells that were pretreated with DMSO (control) or indicated doses of bortezomib for 24 hours. Error bars: standard error of the mean.

a

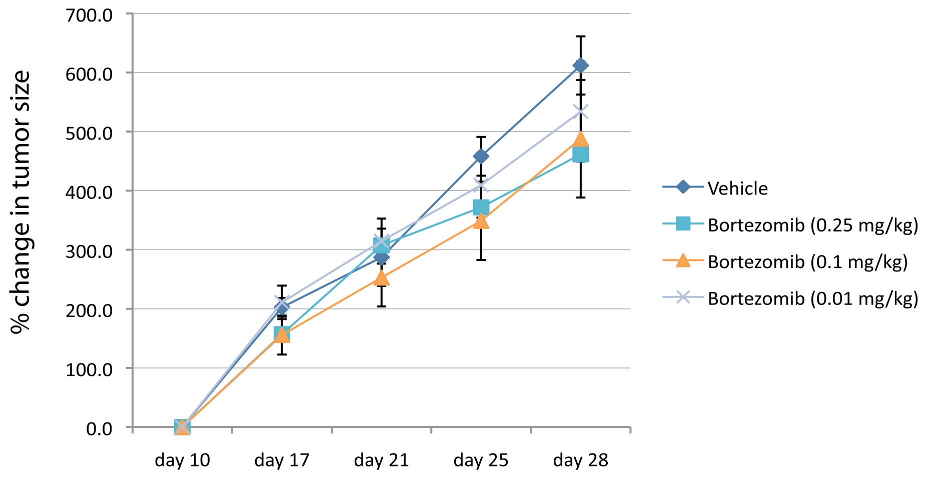

b

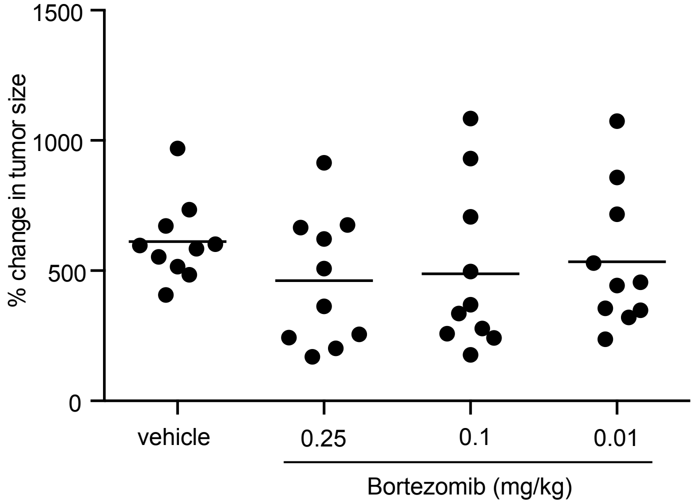

Supplementary Figure 7. Bortezomib alone controls for the xenografts. a, Growth rates of DLD-1 xenograft tumors treated with vehicle and indicated doses of bortezomib once a week for 3 weeks. **b,** % change in volumes of DLD-1 xenograft tumors at the last day of treatment. Bortezomib treated samples were not significantly different than vehicle treated tumors (Mann-Whitney test). Error bars: standard error of the mean.

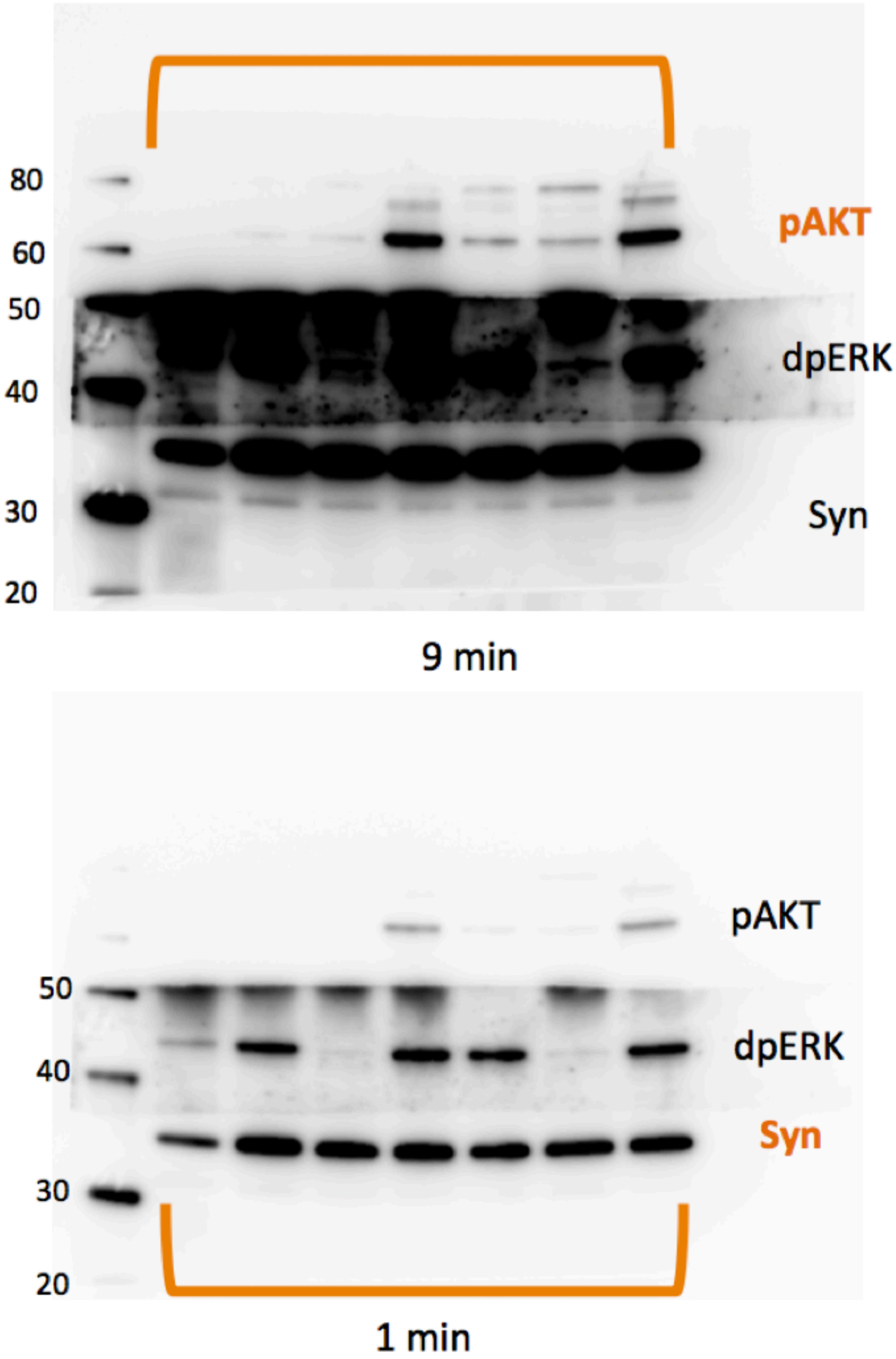

Supplementary Figure 8a: Original blots for pAKT and Syn shown in Figure 6d. Regions indicated in bold orange text and brackets are cropped to generate panel 6d. Images are not altered in any other way.

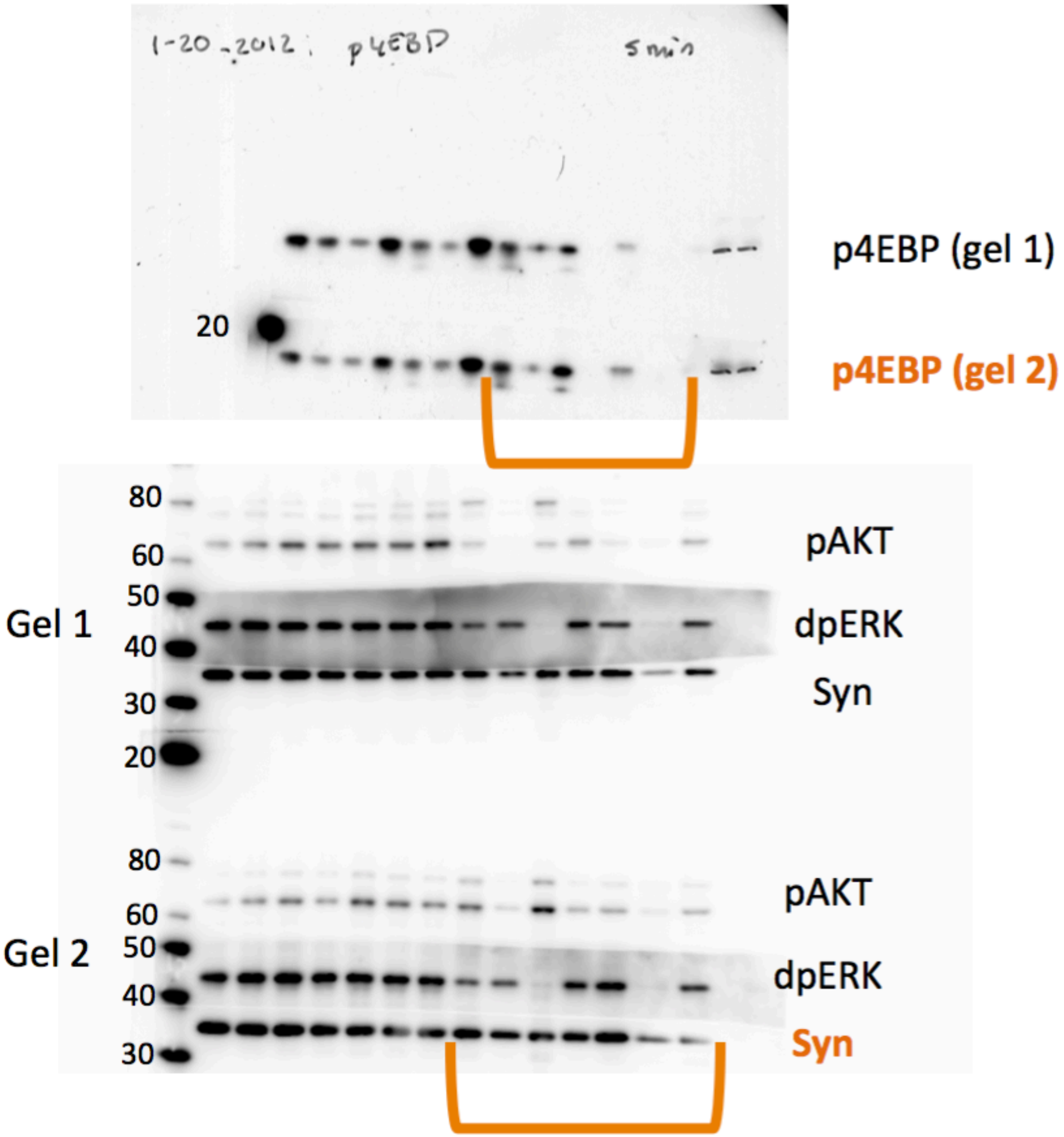

Supplementary Figure 8b: Original blots for p4EBP and Syn shown in Figure 6d. Regions indicated in bold orange text and brackets are cropped to generate panel 6d. Images are not altered in any other way. First 7 lanes that are cropped out are from an unrelated experiment.

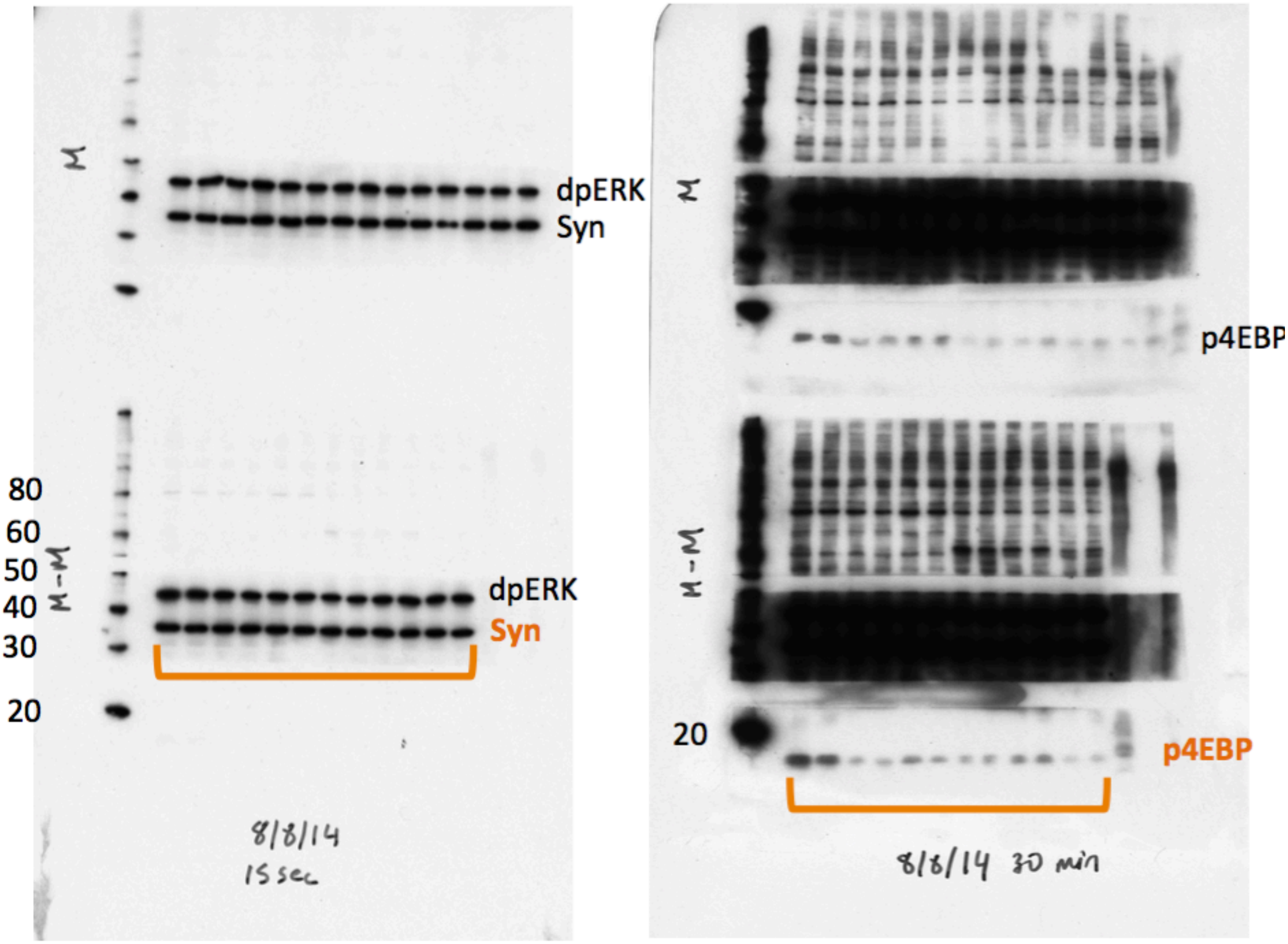

Supplementary Figure 8c: Original blots for p4EBP and Syn shown in Figure 6f. Regions indicated in bold orange text and brackets are cropped to generate panel 6f. Images are not altered in any other way.

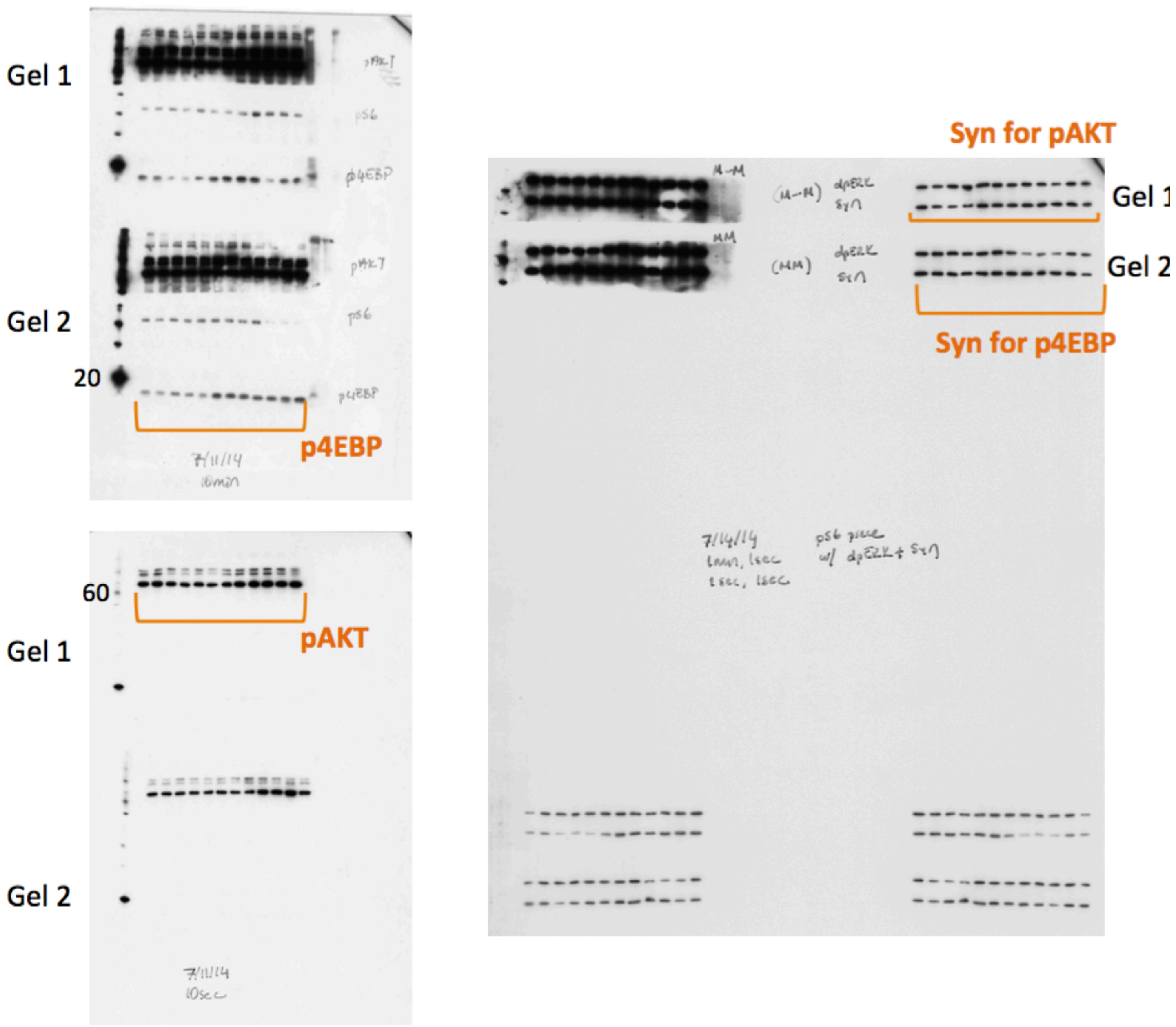

Supplementary Figure 8d: Original blots for p4EBP, pAKT and Syn shown in Figure 7a. Regions indicated in bold orange text and brackets are cropped to generate panel 7a. Images are not altered in any other way.

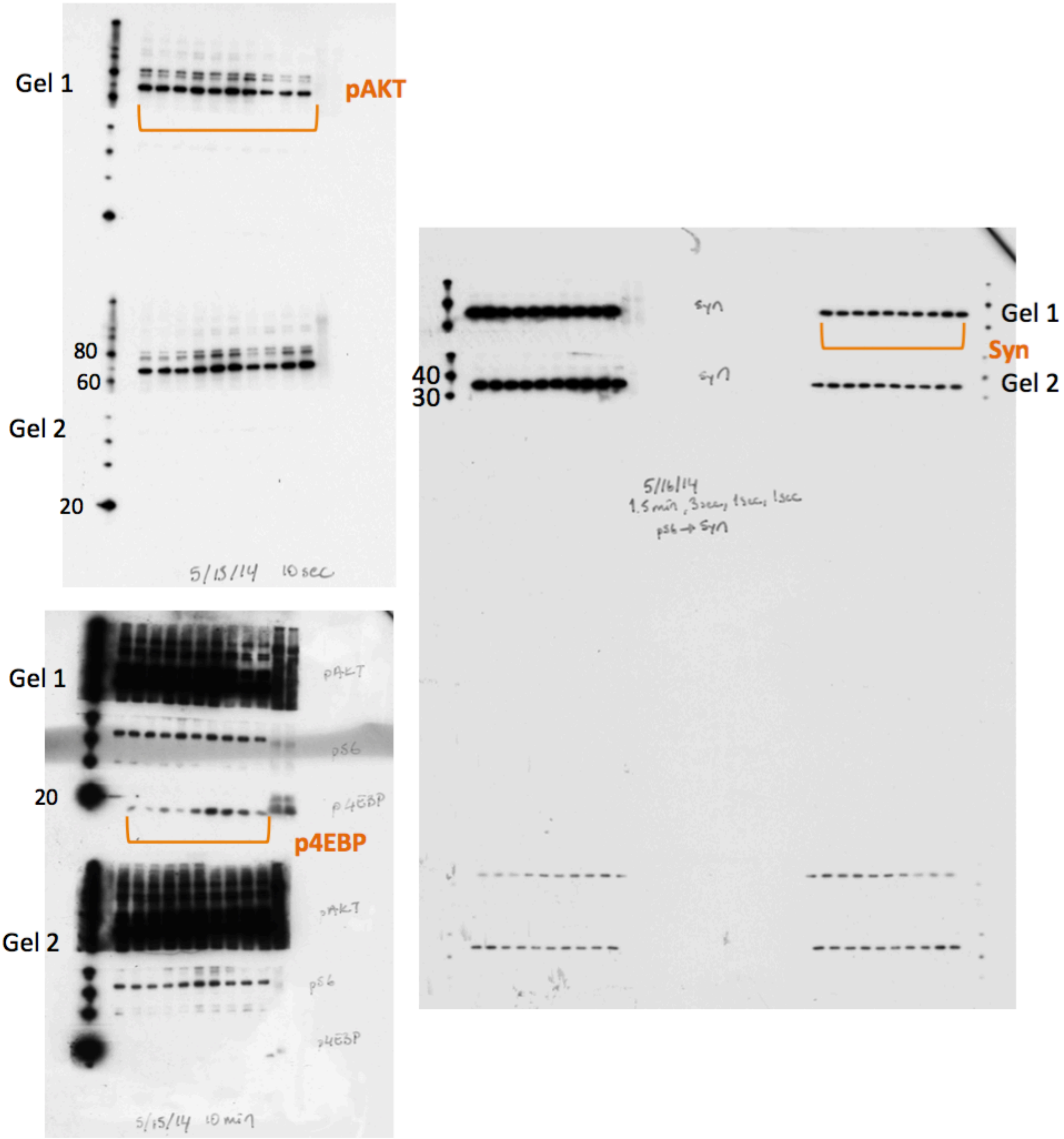

Supplementary Figure 8e: Original blots for p4EBP, pAKT and Syn shown in Figure 8a. Regions indicated in bold orange text and brackets are cropped to generate panel 8a. Images are not altered in any other way.

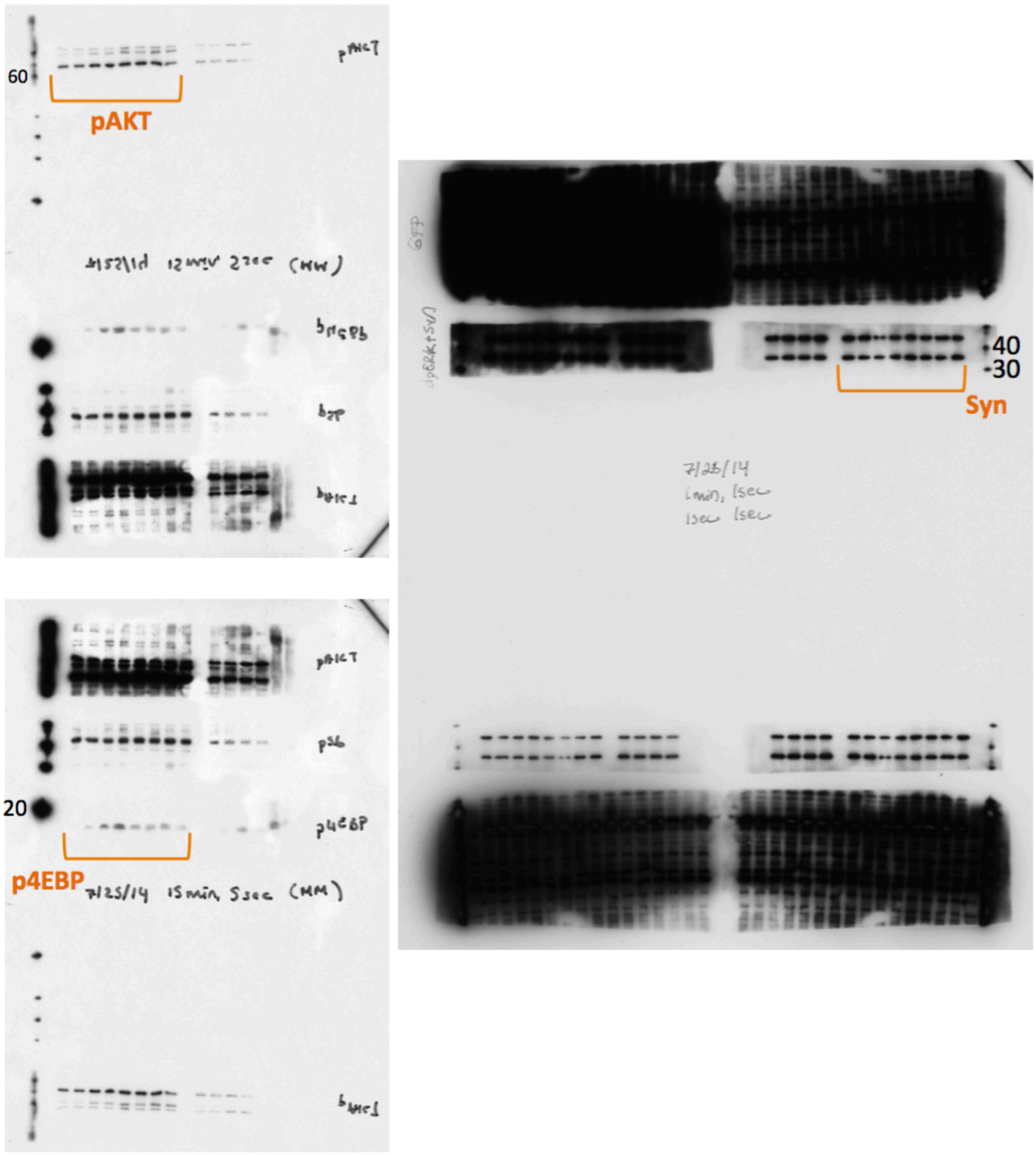

Supplementary Figure 8f: Original blots for p4EBP, pAKT and Syn shown in Figure 8d. Regions indicated in bold orange text and brackets are cropped to generate panel 8d. Images are not altered in any other way.

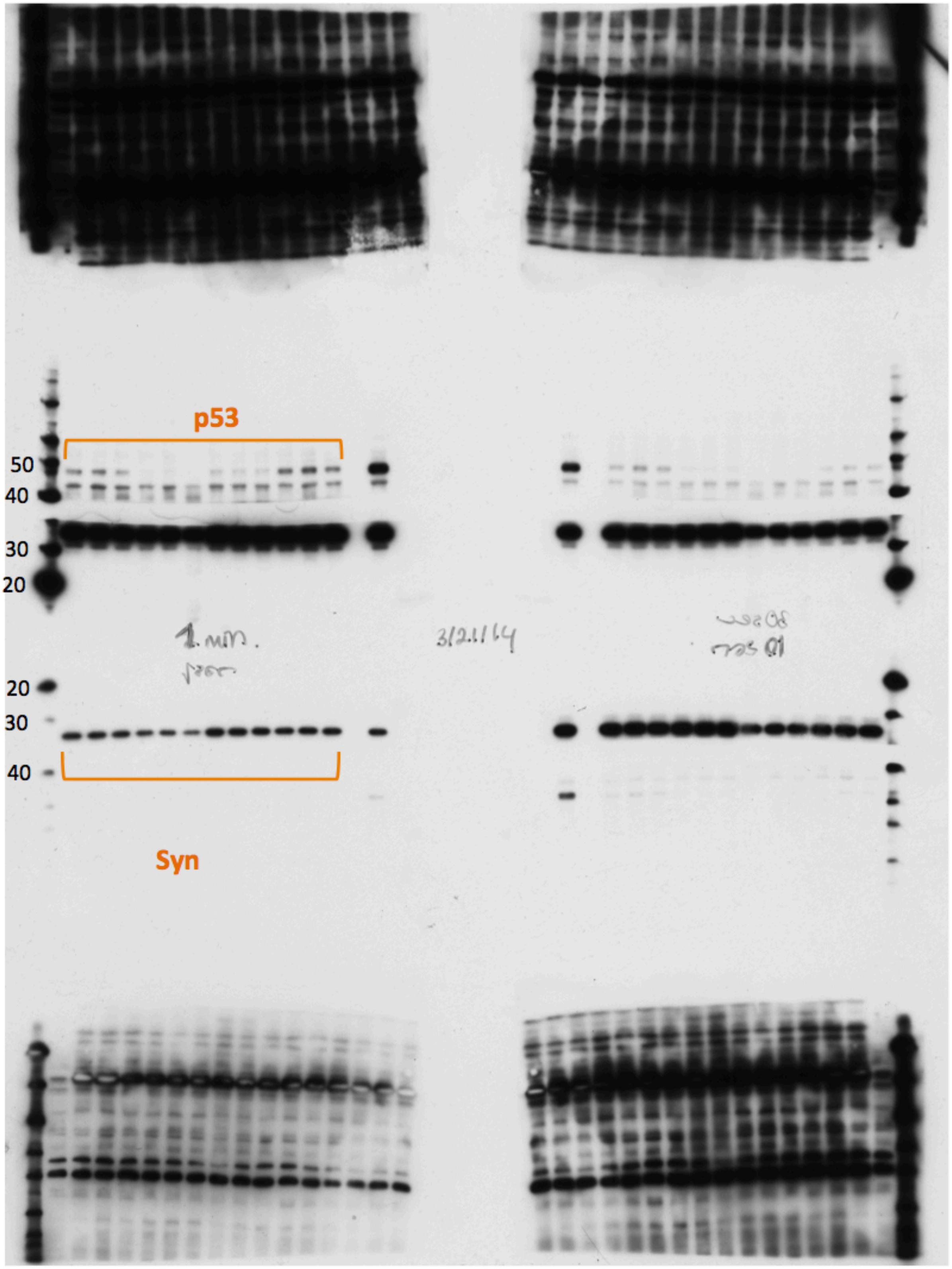

Supplementary Figure 8g: Original blots for p53 and Syn shown in Supplementary Figure 1a. Regions indicated in bold orange text and brackets are cropped to generate the original panel. Images are not altered in any other way. Last lane is from animals overexpressing p53; it is used to confirm the p53 band and is cropped out in the main figure.

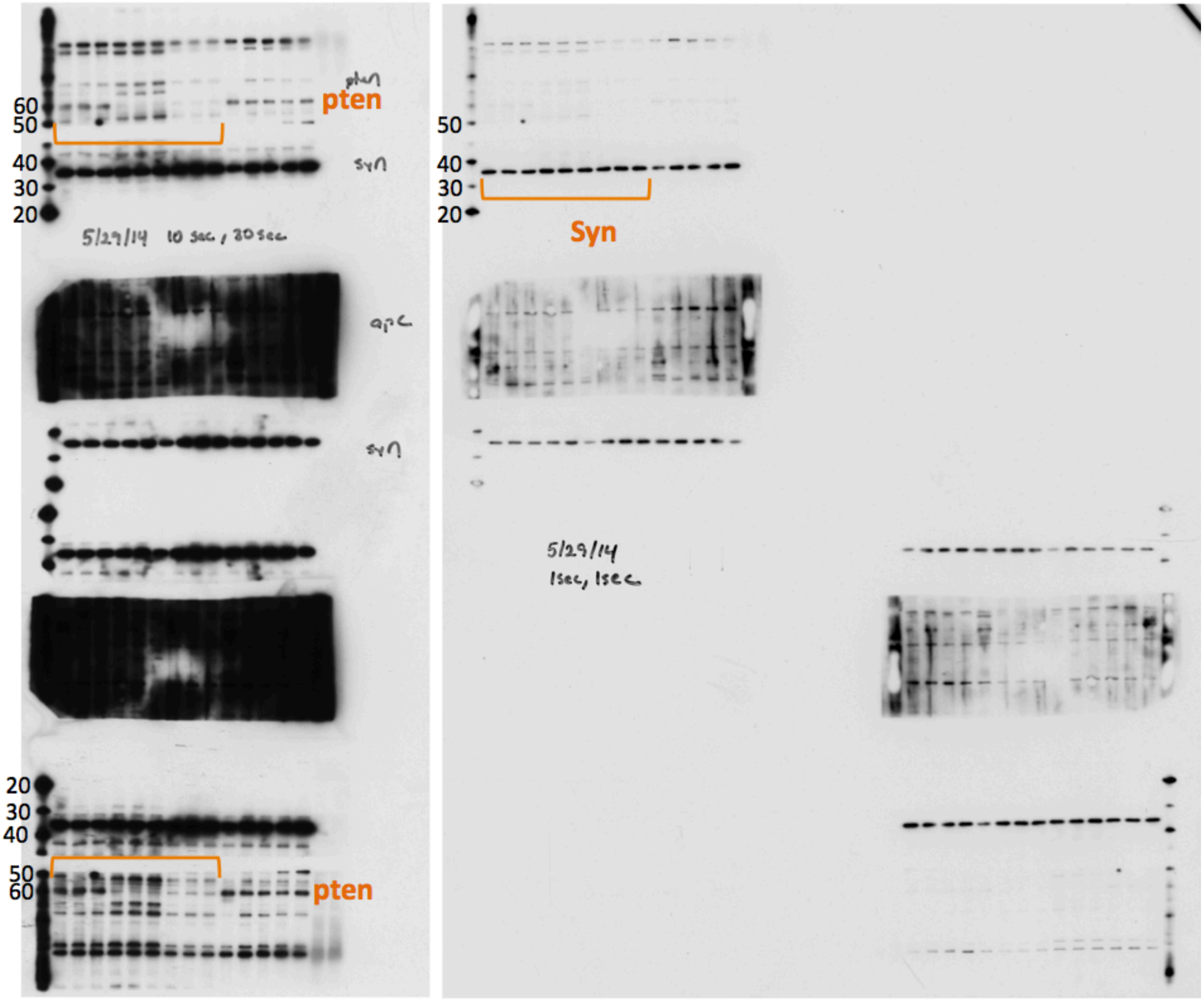

Supplementary Figure 8h: Original blots for pten and Syn shown in Supplementary Figure 1b. Regions indicated in bold orange text and brackets are cropped to generate the panel. Images are not altered in any other way.

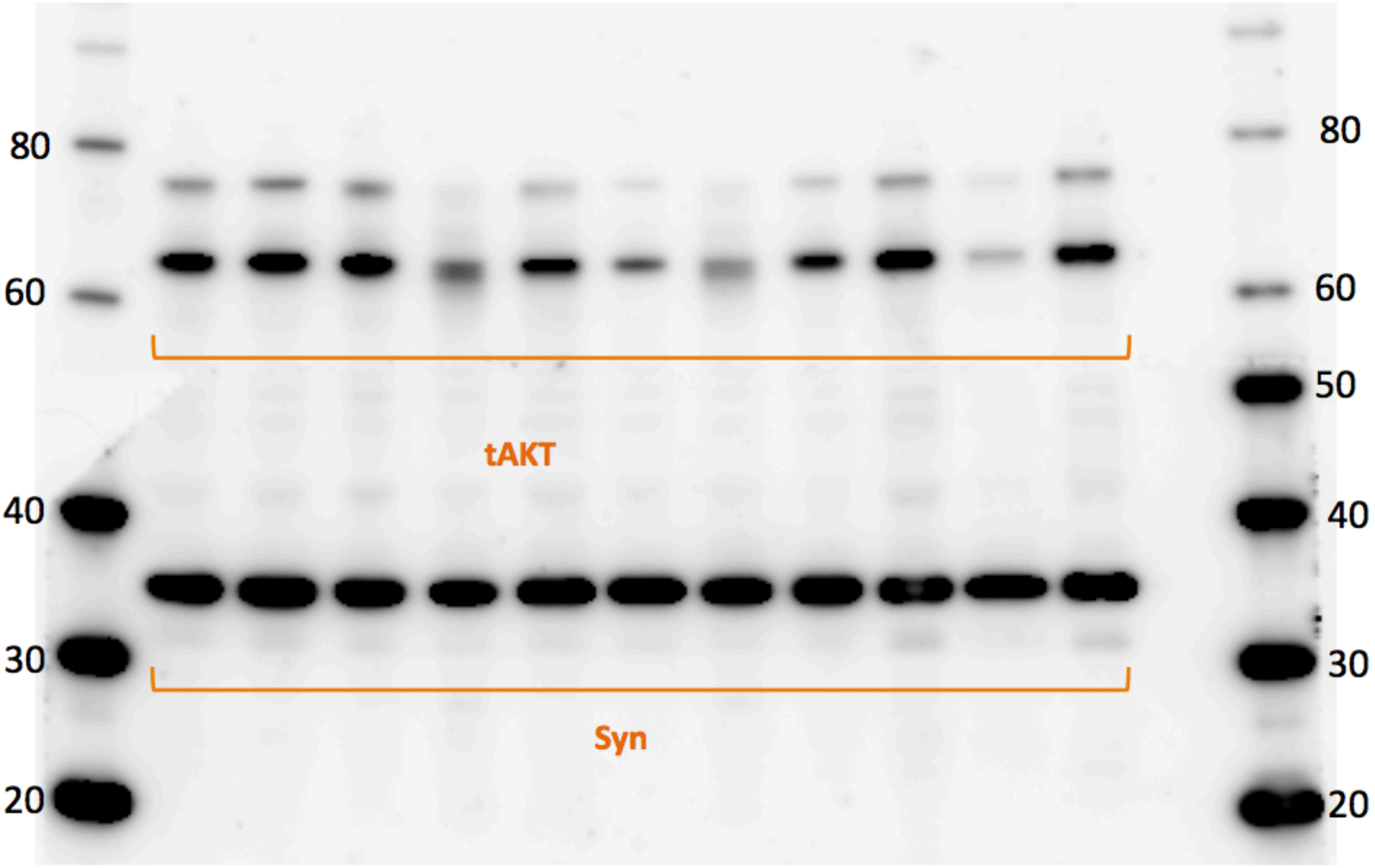

Supplementary Figure 8i: Original blots for total AKT and Syn shown in Supplementary Figure 5. Regions indicated in bold orange text and brackets are cropped to generate Supplementary Figure 5. Images are not altered in any other way.

Supplementary Table 1. Recurrently mutated genes identified by TCGA in colorectal tumors

	GENE_SYMBOL	NUM_CASES_ALTERED	PERCENT_CASES_ALTERED
WNT	APC	162	76%
	TCF7L2	30	14%
	AMER1	24	11%
	AXIN2	14	6%
	CTNNB1	11	5%
	FZD10	1	1%
	**GENE_SYMBOL**	**NUM_CASES_ALTERED**	**PERCENT_CASES_ALTERED**
TGF-beta	SMAD4	29	13%
	ACVR2A	25	11%
	TGFBR2	22	10%
	SMAD2	16	7%
	ACVR1B	16	7%
	SMAD3	9	4%
	TGFBR1	7	3%
	**GENE_SYMBOL**	**NUM_CASES_ALTERED**	**PERCENT_CASES_ALTERED**
RTK/RAS	KRAS	89	42%
	BRAF	22	10%
	NRAS	20	9%
	ERBB2	14	6%
	ERBB3	14	6%
	**GENE_SYMBOL**	**NUM_CASES_ALTERED**	**PERCENT_CASES_ALTERED**
PI3K	PIK3CA	31	14%
	PTEN	13	6%
	PIK3R1	9	4%
	IGF2	5	2%
	IRS2	3	1%
	**GENE_SYMBOL**	**NUM_CASES_ALTERED**	**PERCENT_CASES_ALTERED**
P53	TP53	111	52%
	ATM	26	12%

Supplementary Table 2. Fly models based on TCGA patients

fly models	# of patients	Frequency in the patient population
ras p53 pten dSmad4 apc	10	4.7
**total # of quintuples**	**10**	**4.7**
ras p53 dSmad4 apc	15	7.1
ras pten dSmad4 apc	10	4.7
ras p53 pten apc	5	2.4
p53 pten dSmad4 apc	1	0.5
ras p53 pten dSmad4	0	0.0
**total # of quadruples**	**31**	**14.6**
ras p53 apc	32	15.1
ras dSmad4 apc	13	6.1
p53 dSmad4 apc	12	5.7
ras pten apc	9	4.2
ras p53 pten	6	2.8
p53 pten apc	4	1.9
ras p53 dSmad4	3	1.4
ras pten dSmad4	2	0.9
pten dSmad4 apc	2	0.9
**total # of triples**	**83**	**39.2**
p53 apc	29	13.7
ras apc	16	7.5
ras p53	9	4.2
ras dSmad4	3	1.4
pten apc	3	1.4
p53 dSmad4	2	0.9
p53 pten	1	0.5
dSmad4 apc	1	0.5
**total # of doubles**	**64**	**30.2**
apc	16	7.5
p53	3	1.4
ras	2	0.9
dSmad4	1	0.5
pten	0	0.0
**total # of singles**	**22**	**10.4**
**none**	**2**	**0.9**
